# Spike synchrony as a measure of Gestalt structure

**DOI:** 10.1038/s41598-024-54755-w

**Published:** 2024-03-11

**Authors:** Viktoria Zemliak, Julius Mayer, Pascal Nieters, Gordon Pipa

**Affiliations:** Institute of Cognitive Science, University of Osnabrück, 49074 Osnabrück, Germany

**Keywords:** Computational neuroscience, Dynamical systems, Network models, Neural encoding

## Abstract

The function of spike synchrony is debatable: some researchers view it as a mechanism for binding perceptual features, others – as a byproduct of brain activity. We argue for an alternative computational role: synchrony can estimate the prior probability of incoming stimuli. In V1, this can be achieved by comparing input with previously acquired visual experience, which is encoded in plastic horizontal intracortical connections. V1 connectivity structure can encode the acquired visual experience in the form of its aggregate statistics. Since the aggregate statistics of natural images tend to follow the Gestalt principles, we can assume that V1 is more often exposed to Gestalt-like stimuli, and this is manifested in its connectivity structure. At the same time, the connectivity structure has an impact on spike synchrony in V1. We used a spiking model with V1-like connectivity to demonstrate that spike synchrony reflects the Gestalt structure of the stimulus. We conducted simulation experiments with three Gestalt laws: proximity, similarity, and continuity, and found substantial differences in firing synchrony for stimuli with varying degrees of Gestalt-likeness. This allows us to conclude that spike synchrony indeed reflects the Gestalt structure of the stimulus, which can be interpreted as a mechanism for prior probability estimation.

## Introduction

Neurons in various cortical areas fire synchronously in response to various tasks. First evidence of synchronous firing was found in the sensory cortex, e.g. the primary visual cortex^1^, extrastriate^2^ and primary auditory cortex^3,4^. Additionally, spike synchrony was later discovered in the primary motor cortex^5^ and frontal cortex^6^.

However, the function of spike synchrony has been a subject of a long-standing debate in the neuroscience community. Two most prominent hypotheses argue that synchrony is either a byproduct of brain activity^7,8^, or that it serves as a mechanism of binding the perceived information, thus creating a representation of the whole from the separate input components^9,10^. In the light of this hypothesis, synchrony can be used to perform contour integration^11,12^.

Along with the binding hypothesis, some research suggests that synchrony in the beta or gamma frequency range can serve for attention modulation and relevant stimulus selection^13–15^. Synchrony associated with the attention distribution can also be observed between multiple functional brain areas^16^.

In our work, we argue for an alternative view on the computational role of synchrony: it can indicate the Gestalt structure and hence the prior probability of the incoming visual stimulus^17^. That is, if the cortical units are familiar with the stimulus, they tend to fire synchronously in response to it. We suggest that spike synchrony in populations of neurons is an estimator of the match between the stimulus and the stimulus prior that is encoded in the network structure.

Additionally, we argue that synchrony, and in turn the estimate of the stimulus matching and the encoded prior, is an emergent property that spreads across larger parts of the network. Spike synchrony provides additional information which is not directly encoded in the stimulus pattern.

Last but not least, it is worth mentioning that a number of studies on spike synchrony view it in the context of brain oscillations of various frequencies^18^. However, we want to highlight that we do not rely on oscillatory activity patterns and their frequency or phase in this paper. Instead, we show that the computational role of synchrony arises solely from the noise induced coherence—a mechanism depending on the network connectivity structure.

### Synchrony evidence

Spike synchrony has been observed in various behavioral contexts. In the sensory cortex, synchronous firing depends on properties of the input. For example, neighboring neurons of the primary auditory cortex with similar receptive fields fire simultaneously, which can be seen at all layers between all distinguishable cell types^19^. The synchronous firing of the distant neurons was shown in the primary somatosensory cortex^20^. Spike synchrony has been widely observed in the primary visual cortex (V1). It depends on certain geometrical characteristics of the input stimuli: spatial continuity^1^ and orientation similarity^21^. Thus, neurons of V1 fire synchronously in response to continuous visual stimuli and stimuli with homogeneous angle orientation.

Besides the sensory cortex, synchronous firing was found in the executive areas. In the primary motor cortex, cells with similar muscle fields tend to fire synchronously^22^. There was also evidence that neurons of the primary motor cortex fire synchronously with both the performed and the intended action^23^. The results of the latter might be perceived controversial due to the choice of the analysis method, but they were replicated with use of the more robust techniques^24^.

Synchronous firing can also correlate to higher cognitive functions. Cells in the middle temporal area (MT) fire synchronously in relation to decision making^25^. In the prefrontal cortex, the neurons can exhibit simultaneous firing during working memory tasks^26,27^. The firing of the neurons of the inferotemporal cortex can synchronize when monkeys process face features^28^.

### Neural basis of synchrony

The neural basis of spike synchrony lies in horizontal intracortical connections^29,30^. These connections mostly arise from pyramidal cells and are parallel to the cortical surface^31^. Their important property is that neurons which fire in similar contexts tend to form such strong connections. Horizontal connections were found in various brain areas, e.g. in the primary motor cortex between neurons representing similar muscle groups^32^.

The sensory cortex was also shown to be full of intracortical horizontal connections. For example, cells of the auditory cortex that have similar bandwidth and characteristic frequency selectivity are well connected to each other^33^. Horizontal connections in the somatosensory cortex can be formed even between the distant cells which respond to tactile stimuli at the opposing fingertips^34^. Interestingly, in this case well connected cells of the somatosensory cortex do not have similar receptive fields, but rather are engaged in the common somatosensory context.

Since the current research is primarily focused on the primary visual cortex, we describe its horizontal connections and other architectural properties in more detail. V1 area is known to be retinotopically organized, i.e. its neurons respond to the stimuli, whose retinal coordinates match the cortical position of these neurons. Besides, these neurons have orientation selectivity. Thus, each V1 cell is spatially and orientationally tuned: it fires when there is a visual stimulus with the particular angle in the particular region of the retina. The cells that are responding to the specific orientation are forming cortical columns, which are in turn organized in hypercolumns. Columns that constitute one hypercolumn share the similar receptive fields^35^.

There are two main factors that influence presence and strength of the horizontal intracortical connections in V1: the spatial proximity of the receptive fields, and the similarity of the preferred orientation^36,37^. Thus, the cells that are responding to spatially neighboring visual stimuli of the similar orientation, tend to have strong horizontal connections. On the physiological level, the stronger connections between neurons with similar orientation tuning are manifested in larger synapses^38^.

At the same time, intracortical horizontal connections are subject to change, learn, and adapt to the experience. In adults, the plasticity of horizontal intracortical connections is associated with acquiring new skills^39^. During the development of the visual cortex, first the unclustered and weakly specified horizontal connections arise. Later they become more refined and fine-tuned under the control of visual experience^40,41^.

### Natural image statistics and Gestalt principles

Thus, the horizontal connectivity in V1 reflects the acquired visual experience. This experience can be manifested in the form of aggregate statistics of experienced natural visual stimuli^42^. There has been numerous research on various environmental statistics that can influence neuronal connectivity and neuronal activity patterns (see Ref.^43^ for review). So, what are the relevant statistics of natural visual scenes that shape the horizontal connectivity in V1?

Brunswik & Kamiya have first shown that one of the crucial statistical bases in natural images are the Gestalt principles^44^. The Gestalt principles describe the mechanism for grouping and interpreting visual elements. The elements which are following these principles are more likely to be perceived as a holistic object, rather than separate ones. The Wertheimer landmark paper included six Gestalt principles: proximity, similarity, uniform density, direction, common fate, good continuation^45^. Since then, the Gestalt theory has been highly debatable, and various researchers have suggested up to a hundred new grouping principles^46^. On the contrary, there is an idea that all Gestalt principles are the special instances of a single general Good Gestalt principle^47^.

The natural images statistics have been shown to be consistent with certain Gestalt principles, including proximity, continuity and similarity^44,48–51^. It was also demonstrated how the Gestalt-based perceptual grouping can be shaped and modulated by the visual experience^52,53^. Sigman et al. found that the regularities in natural images follow the Gestalt continuity principle, and showed how such regularities are reflected in the V1 connectivity^51^. In Onat, Jancke, & König the Gestalt principles were also shown to be manifested in the V1 connectivity, but with the natural movies used as stimuli, rather than static images^42^.

As summarized in Korndörfer et al. most often experienced and behaviorally relevant natural visual stimuli appear to have the Gestalt-like structure, and it manifests at the level of intracortical connectivity^17^. And this Gestalt-structured connectivity, in turn, is assumed to be the basis of spike synchrony in visual cortex^29–31,36^.

Also, Ref.^17^ suggested the mechanism behind spike synchrony emerging in the system in the presence of noise. They showed that the noise-induced coherence is dependent on the connectivity structure and the connectivity strength, and is reflecting a match between the input stimuli and connectivity patterns encoded in the network. In our study, we use this mechanism by designing specific connectivity patterns we see in V1, to study several Gestalt principles known from psychophysics: proximity, similarity, and continuity (see “Results” for the description of said principles and corresponding experiments).

## Results

**Figure 1:**
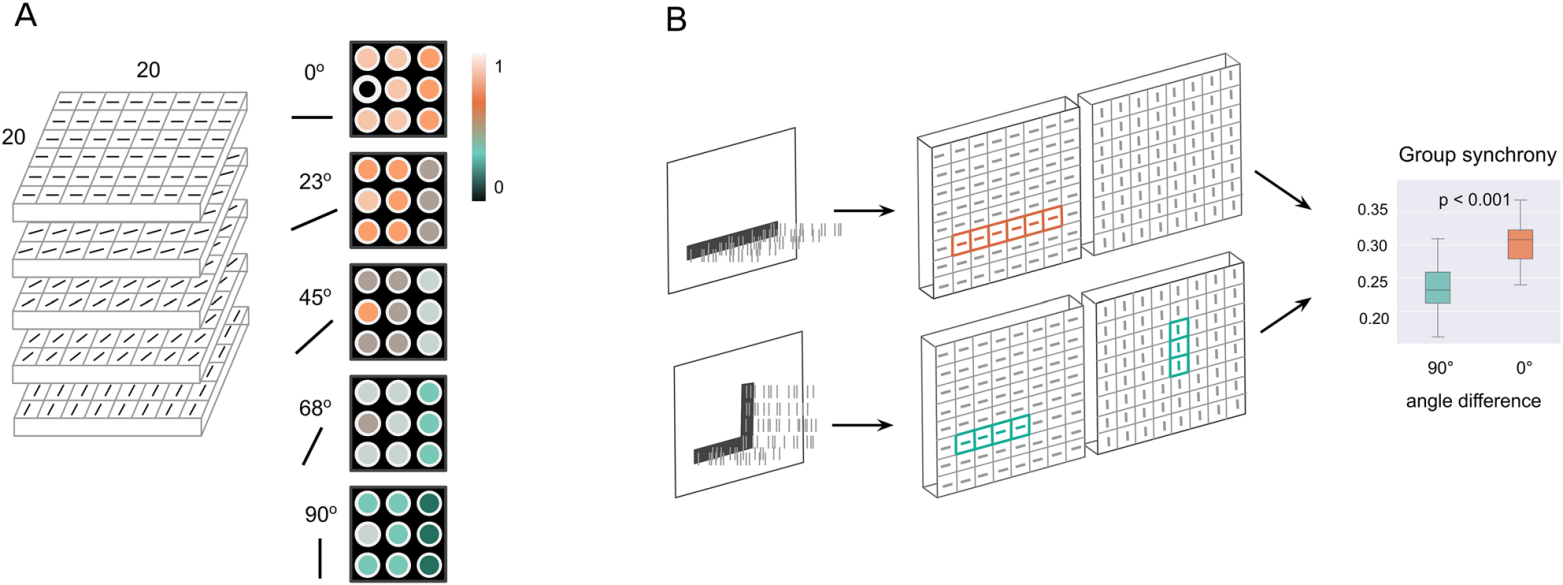
Model and simulation pipeline. (**A**) Model architecture: neurons with spatial and angle preferences, with specified connections. All connections of a black neuron with neurons in a neighborhood of 2 are shown. A color bar shows the relative connectivity strength. (**B**) Simulation pipeline: stimuli patterns of various Gestalt-likeness are sent to the model as Poisson spike trains; spatially- and orientation-selective neurons in the model detect angles and become activated in the locations which receive input; spike synchrony is measured across the neurons from the activated subsets.

We used a pulse-coupled fully-excitatory Izhikevich spiking network^54^ in a noisy environment, to show that neuronal synchrony reflects how closely the visual input stimulus follows the Gestalt principles. Our network consists of Izhikevich neurons with additional dynamics of open receptors and neurotransmitter conductances (see “Methods” for a detailed model description). Neurons in the model are organized retinotopically, have spatial and orientation selectivity, and are connected in all-to-all fashion. The schematic model representation is depicted in the left panel of Fig. 1.

The connectivity structure is defined by a connectivity matrix and follows a simple rule: the connection strength between two neurons depends on their spatial distance and difference in orientation selectivity. Thus, neighboring neurons with shared orientation preferences are strongly connected, whereas the most distant neurons with different orientation selectivity form the weakest connections. The preferential connections and larger synapses between neurons with similar receptive fields were shown in V1^36,38^.

Thus, each neuron receives lateral input through the horizontal connections defined in a connectivity matrix, and external input from the input images. We simulated 2 s of activity of each neuron. We measured the synchrony of their firing responses as the noise-induced coherence, and refer to it as Rsync (see “Methods”).

To study how spike synchrony reflects the Gestalt structure of stimuli, we ran three experiments for three Gestalt principles: proximity, similarity, and continuity. Within each experiment, we first generated a set of artificial input stimuli, then ran 100 simulation trials of our model on each stimulus (14 stimuli for all experiments in total × 100 trials). One run of every trial simulated 1500 ms of neuronal activity. Finally, for each input stimulus, we measured and averaged firing synchrony over all 100 trials. Figure 1 shows the experimental pipeline.

For each simulation experiment, we used a set of stimuli that followed the Gestalt principles to a different degree. In other words, stimuli had gradual Gestalt-likeness. One stimulus consisted of several line segments, which were organized in greater or lower accordance with a certain Gestalt principle (see Fig. 8 in "Methods").

1. The Gestalt principle of *proximity* implies that single visual items near each other are perceived as an aggregation into a larger visual composition. In our context, that refers to input stimuli consisting of two segments on various distances from each other. The smaller the distance between the segments, the more Gestalt-like the entire stimulus.
2. Visual components can also be integrated into perceptual groups based on the similarity of their appearance. This classification of elements by visual features is an instance of the Gestalt principle of *similarity*. In our work, we interpret similarity as shared angle orientation: if two parts of the stimulus share the similar orientation, the entire stimulus is highly Gestalt-like. Hence, the bigger the angle difference, the less Gestalt-like the stimulus is.
3. The Gestalt principle of *continuity* is based on the idea that visual elements are preferably grouped if they are organized in a continuous line. Thus, elements with abrupt changes in orientation are more likely to be perceived as separate entities. In our experiments, each continuity stimulus consists of 4 segments. Segments 1 and 3 share the same orientation and thus constitute a continuous line, and segments 2 and 4 share another orientation. The smaller the orientation difference between these two continuous lines, the more the entire stimulus follows a Gestalt principle.

Essentially, the input stimuli for similarity consist of two lines making contact at a single point, while the continuity stimuli consist of two lines crossing each other at the intersection point. The proximity stimuli are represented by two lines of the similar angle orientation, located on various distances from one another (see “Methods” for a detailed stimuli description).

We measured synchrony of firings of neurons in the model, in response to input stimuli. Importantly, we considered only those neurons which were receiving external input from the stimuli. The input images were used as a model input in the form of the Poisson spiking process. Please see “Methods” for a comprehensive description of every stimulus, and the procedure of transforming image stimuli into Poisson spiking input.

**Figure 2:**
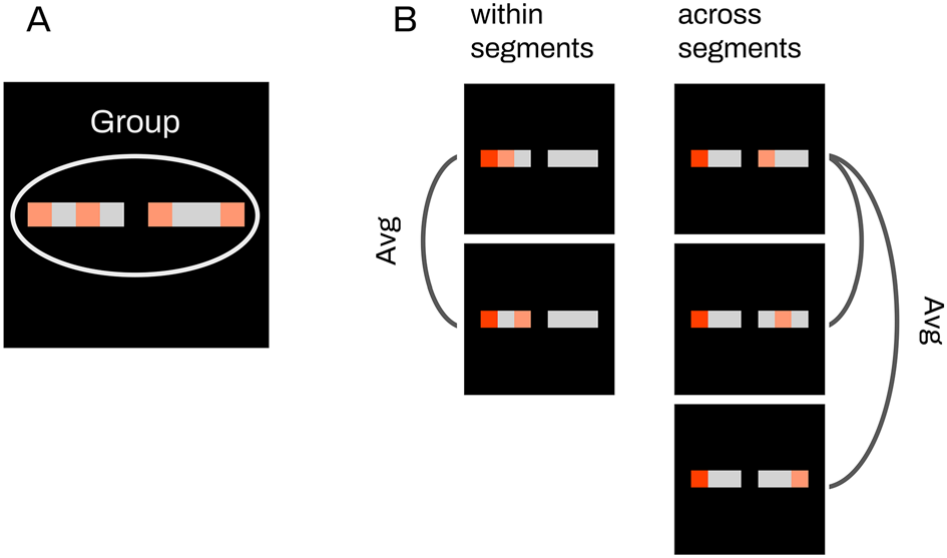
Selecting neurons for synchrony measurements. (**A**) Group Rsync. Several neurons are selected from each stimulus segment, then synchrony is measured for the group of all selected neurons (marked orange in the picture). (**B**) Avg Pairwise Rsync. For each neuron (consider the red neuron as an example), we first measured its firing synchrony with every other neuron from the same segment (marked orange) and averaged the result. Second, we measured its synchrony with every other neuron from the other segment (marked orange) and averaged this result too.

Our main measurement was Group Rsync, and we additionally measured Rsync in pairwise fashion (we further refer to it as Avg Pairwise Rsync), to better illustrate the differences visually. Figure 2 provides an example of selecting neurons for both measurements.

For Group Rsync, we randomly selected 4 neurons from each stimulus segment and measured the synchrony of all their firings together. The amount of neurons to measure within one segment is arbitrary, we tested various numbers from 1 to 6 and did not observe any difference in the results. For Avg Pairwise Rsync, we measured and then averaged synchrony between all pairs of neurons from similar and different segments of the stimuli independently.

### Synchrony for minimal and maximal Gestalt-likeness

**Figure 3:**
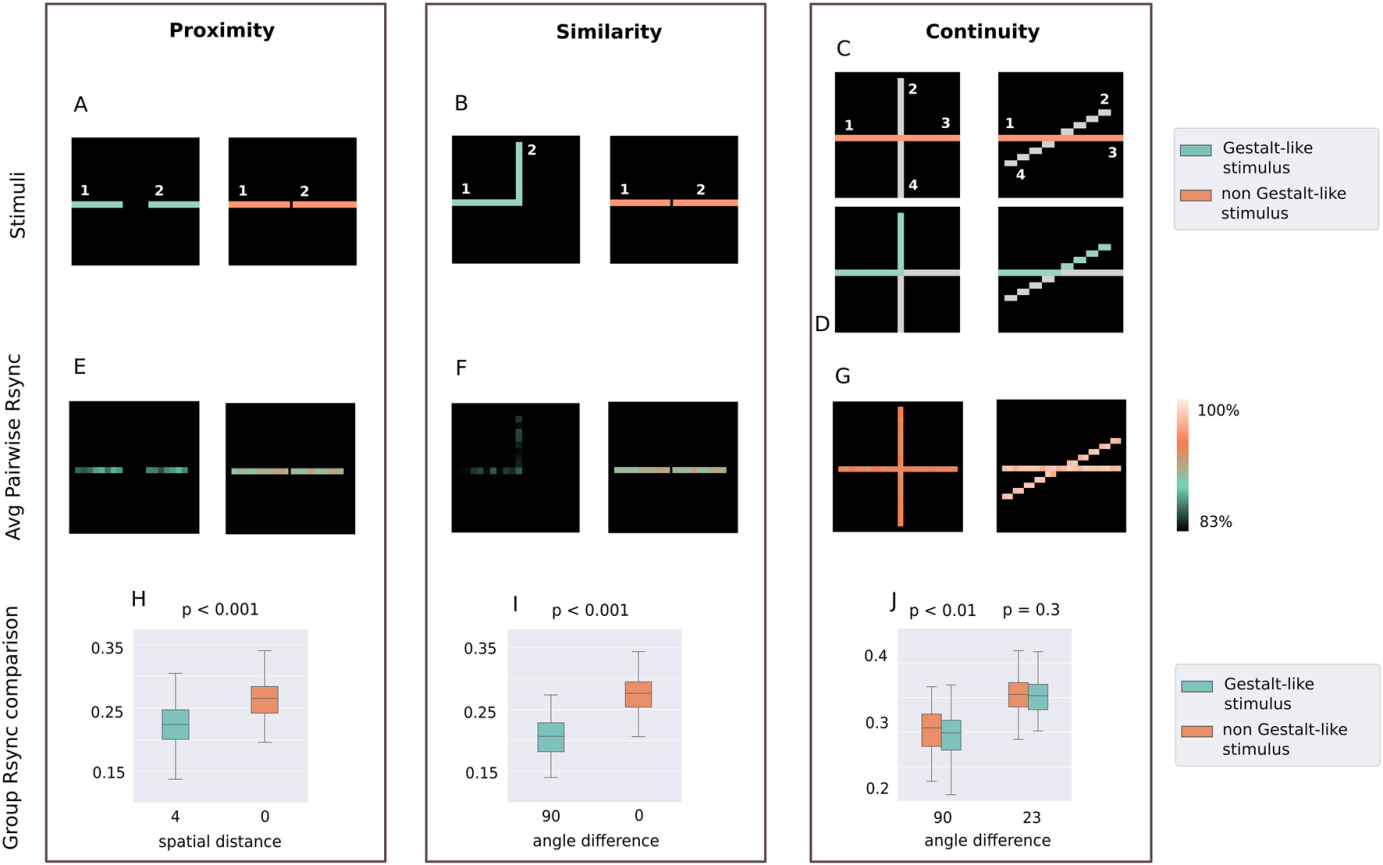
Rsync differences for most Gestalt-like and least Gestalt-like stimuli. (**A**–**D**) Stimuli for proximity, similarity, and continuity are described in further detail in Methods. (**E**–**G**) Average pairwise Rsync for each neuron’s spike train with every neuron from the other segment. Shown in the percentage from maximal measured Rsync. (**H**–**J**) Group Rsync was measured within a group of 10 randomly selected neurons, 5 for each stimulus segment.

First of all, we measured the synchrony between firings of neurons receiving input from two different line segments (Fig. 3). For the proximity and similarity principles, we considered left and right segments. For the continuity principle, the synchrony was measured between neurons which receive input from pairs of segments: left and top, left and right, right and bottom, top and bottom. From each segment, 5 neurons were randomly selected for synchrony measurements.

Measured synchrony reflected how closely the entire stimulus was following a certain Gestalt principle. Highly significant (p < 0.001) differences in synchrony were detected between stimuli with the greatest and the lowest Gestalt-likeness for the proximity and similarity principle, and significant difference (p < 0.01) for the continuity principle. We further report the results for all three principles.

For the proximity principle, the difference was measured as the size of the gap between two segments. The smaller the gap, the stronger the entire stimulus was following the proximity principle. Here, we considered stimuli under two conditions: minimal (0 pixels) and maximal (4 pixels) distance between two segments. The median synchrony was equal to 0.26 between two segments of the stimulus with a spatial distance equal to 0 (min. spatial distance). The median synchrony was equal to 0.22 for segments of the stimulus with the spatial distance equal to 4 (max. spatial distance). The non-parametric Wilcoxon rank test showed a significant difference with the p-value being less than 0.001.

For the similarity principle, the angle orientation difference between two line segments was measured. The smaller the difference, the closer the entire stimulus was following the Gestalt principle of similarity. We considered stimuli under two conditions: minimal (0°) and maximal (90°) angle difference between two segments. The median synchrony was equal to 0.28 between two segments of the stimulus with an angle difference equal to 0° (both segments having equal angle orientation). For the stimulus with a between-segment angle difference equal to 90°, the median synchrony was only 0.2. The Wilcoxon rank test showed a significant difference with the p-value being less than 0.001.

For the continuity principle, the angle difference between two lines was measured, with a smaller difference indicating that the stimulus was following the continuity principle more strongly. We considered stimuli under two conditions: minimal (23°) and maximal (90°) angle difference between two lines. For the largest angle difference (90°), the median synchrony between segments lying on the same line was equal to 0.306. The median synchrony between segments of different lines was equal to 0.299. The Wilcoxon rank test showed a significant difference with the p-value equal to 0.003. As for the smallest angle difference (23°), the median synchrony between segments of the different lines was equal to 0.353. The median synchrony between segments of the same line was equal to 0.355. The Wilcoxon rank test showed a non-significant difference with the p-value equal to 0.34. Thus, the difference in synchrony was more pronounced for a larger difference between the two lines. For the smallest difference (23°), the difference in synchrony was insignificant.

The difference in synchrony between more and less Gestalt-like stimuli was more prominent for the proximity and similarity experiments, compared to the continuity experiment. At the same time, the overall synchrony was greater for both continuity experiment setups (23° and 90° angle difference) than for any of the others. The maximal measured synchrony for the continuity experiments exceeded 0.4, while the maximal synchrony for proximity and similarity was under 0.35. Such inconsistency can be explained by the greater overall excitation in the continuity experiments, since more neurons were receiving both external input and lateral input from the horizontal connections. We assume that lateral input is crucial for establishing synchrony, since neurons have to interact with each other in a recursive fashion through the synapses. However, all the experiments with varying significance levels and effect sizes showed that the Gestalt-likeness of the input is reflected in the spiking synchrony of corresponding neurons.

Throughout the experiments we assumed that spike synchrony highly relies on the temporal structure of the spike trains, rather than non-temporal characteristics such as firing rate. To test this assumption, we did a control measurement for all three experiments. For each spike train, spike times were randomly jittered across the time scale. No differences in synchrony were observed between Gestalt-like and non Gestalt-like conditions on time-jittered data (see Supplementary Note 1).

### Group synchrony and the Gestalt structure of the stimulus

We tested how spike synchrony changes with respect to gradual changes in the Gestalt structure of the stimuli. For the Gestalt principles of proximity and similarity, synchrony increases gradually alongside the increasing Gestalt-likeness of the stimuli. For continuity, the difference in synchrony between Gestalt-like and non-Gestalt-like stimuli segments also increases simultaneously with the increasing difference between these segments.

**Figure 4:**
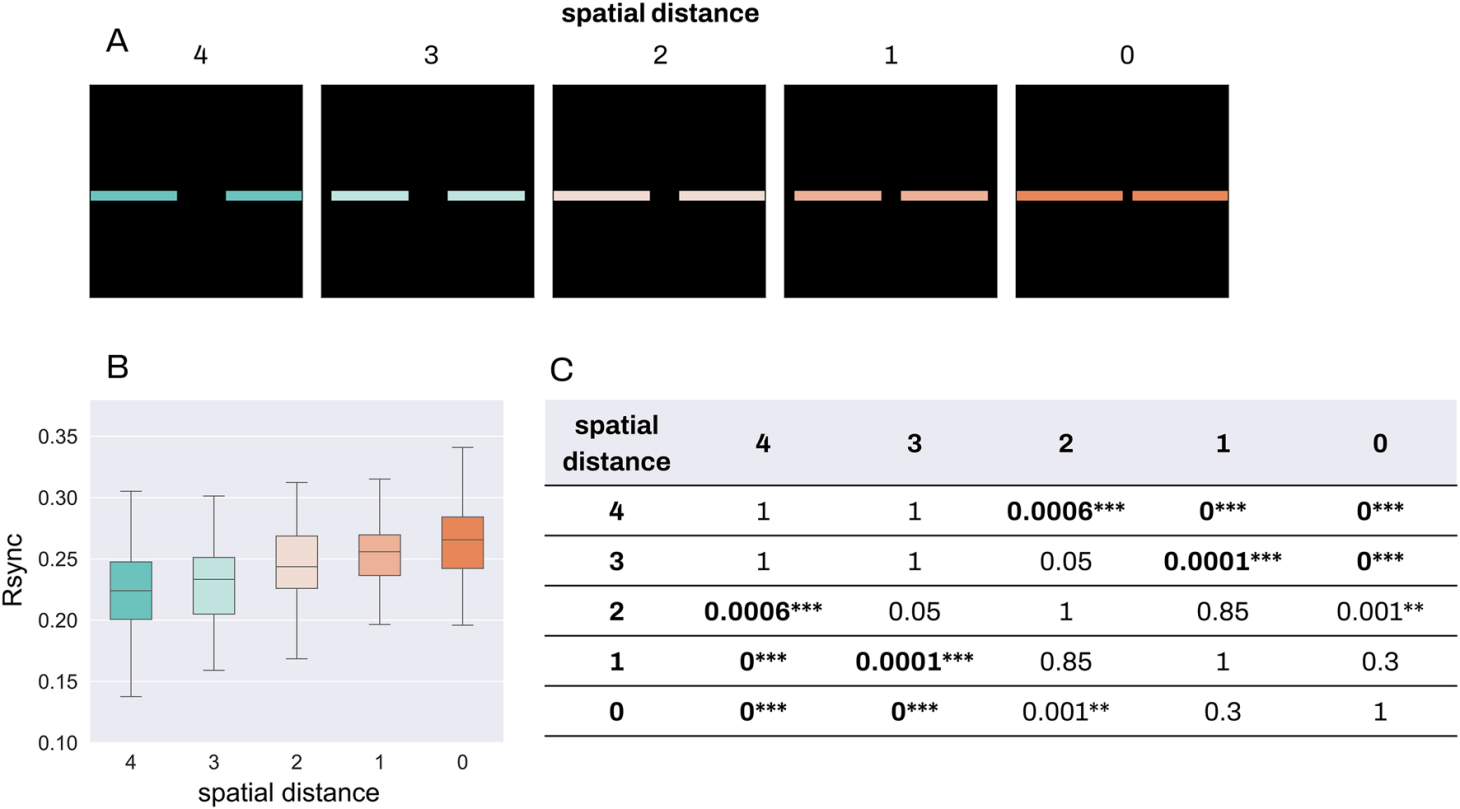
Group Rsync for proximity. Comparison of group Rsync for all stimuli conditions in the proximity experiment. For every stimulus, 4 neurons were randomly selected from each stimulus segment, and the group Rsync was measured between all 8 of them. (**A**) Proximity stimuli with various distances between the segments. (**B**) Comparison of group Rsync for all the stimuli. (**C**) Results for non-parametric Dunn test with Bonferroni adjustment for multiple comparison, rounded to 4 decimal. * stands for p < 0.05, ** for p < 0.01, *** for p < 0.001.

In the proximity experiment, we implemented the Gestalt-likeness of the stimulus as varying spatial distances between the segments: as the spatial distance increases, the Gestalt-likeness decreases (Fig. 4). We used input stimuli with between-segment distances varied from 4 to 0 and measured group spike synchrony for each stimulus.

Kruskal–Wallis test showed significant between-group differences with p-value < 0.0005, and the effect size equal to 0.22. We further applied nonparametric Dunn test with Bonferroni adjustment for multiple comparisons and found statistically significant differences between the following groups: distances 0 and 2 (p < 0.01), 0 and 3 (p < 0.001), 0 and 4 (p < 0.001); 1 and 3 (p < 0.001), 1 and 4 (p < 0.001); 2 and 4 (p < 0.001). Interestingly, differences for distances 1 and 2, 2 and 3, 3 and 4 were not significant. The most Gestalt-like stimulus with the 0 distance between segments led to significant differences in synchrony with the stimuli with all the other between-segment differences. The most significant differences were observed between stimuli with the greatest (0 distance) and lowest (3 and 4 distance) Gestalt-likeness.

**Figure 5:**
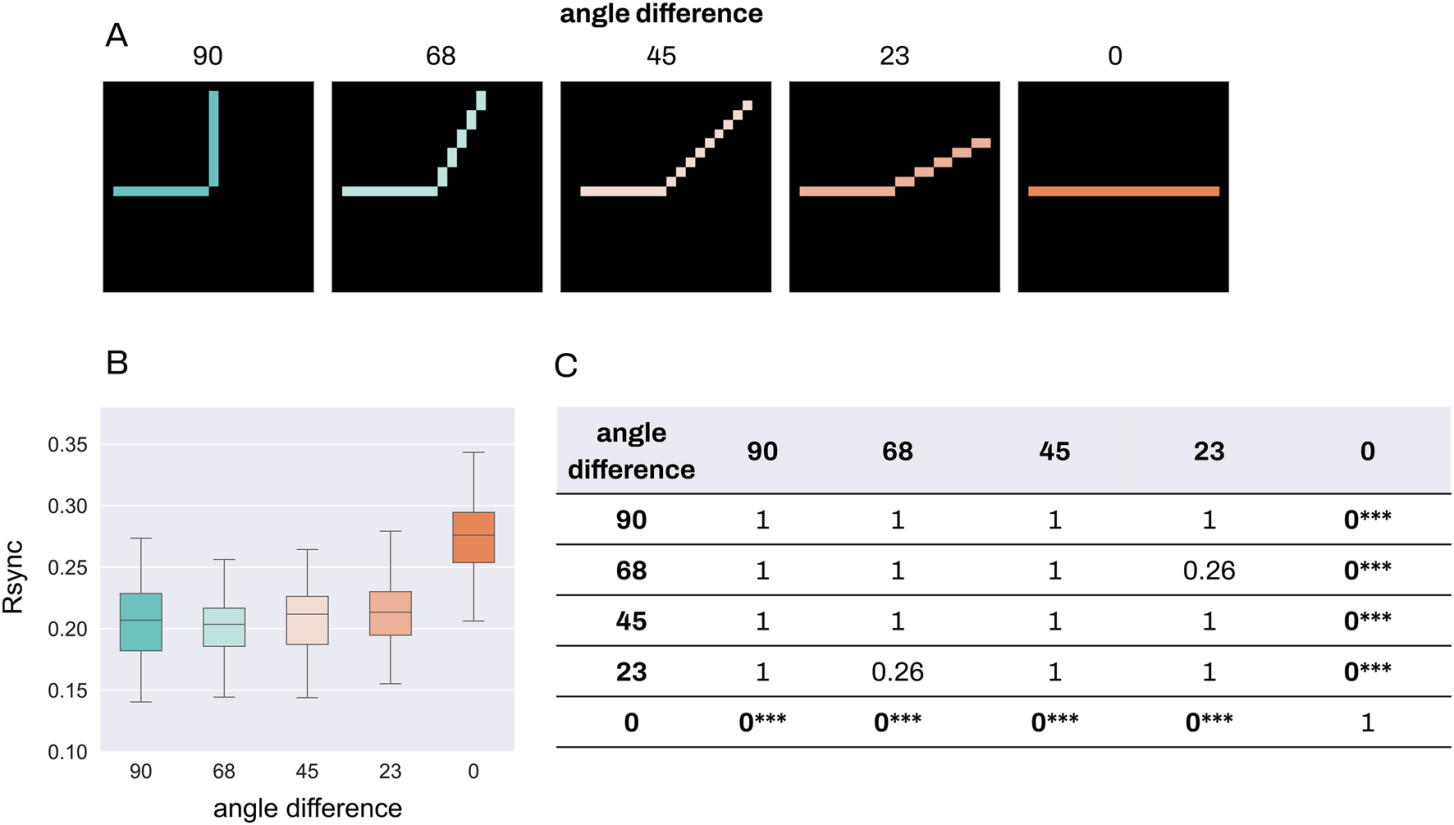
Group Rsync for similarity. Comparison of group Rsync for all stimuli conditions in the similarity experiment. For every stimulus, 5 neurons were randomly selected from each stimulus segment, and the group Rsync was measured between all 8 of them. (**A**) Similarity stimuli with various angle differences between the segments. (**B**) Comparison of group Rsync for all the stimuli. (**C**) Results for non-parametric Dunn test with Bonferroni adjustment for multiple comparison, rounded to 2 decimal. *** stands for p < 0.001.

In the similarity experiment, the angle difference between the stimulus segments was designed to reflect its Gestalt-likeness: a smaller difference corresponds to higher Gestalt-likeness (Fig. 5). Similar to the proximity experiment, we used input stimuli with various angle differences from 90° to 0° and measured the group spike synchrony.

Kruskal–Wallis test showed significant between-group differences with p-value < 0.0005, and an effect size equal to 0.3. The subsequent non-parametric Dunn test with Bonferroni adjustment for multiple comparisons showed significant differences between the following groups: angle differences 0° and 23° (p < 0.001), 0° and 45° (p < 0.001), 0° and 68° (p < 0.001), 0° and 90° (p < 0.001). Only the most Gestalt-like stimulus with the 0° angle difference between segments led to highly significant differences in synchrony with the stimuli with all the other between-segment differences.

**Figure 6:**
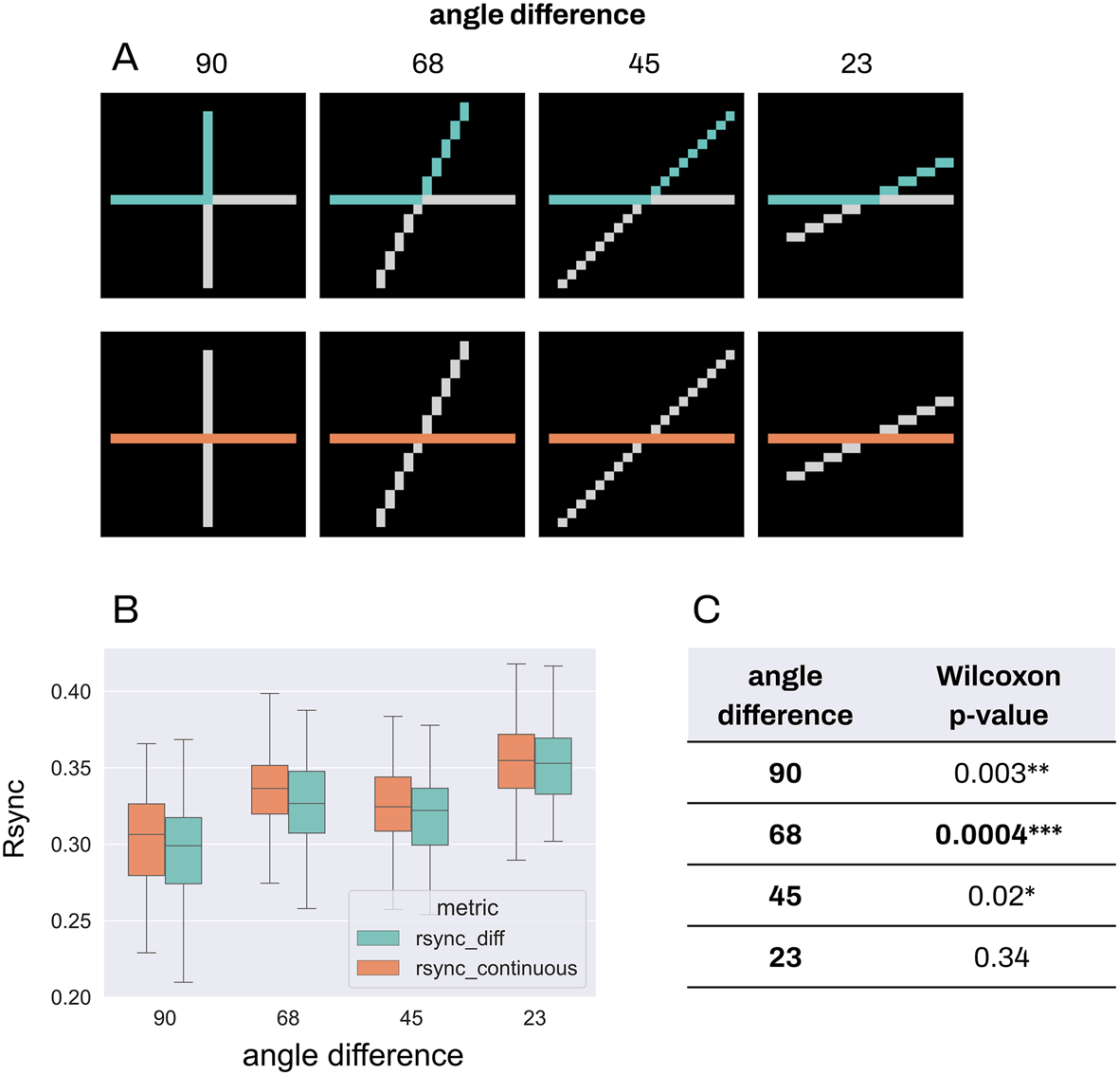
Group Rsync for continuity. Comparison of group Rsync for all stimuli conditions in the continuity experiment, for neurons from continuous and different segments of the stimuli. For every stimulus, 5 neurons were randomly selected from each stimulus segment, and the group Rsync was measured between all 10 of them. Please see Fig. 8 in Methods for details on stimuli segmentation. (**A**) Continuity stimuli with various angle differences between the segments. (**B**) Comparison of group Rsync between different and continuous segments for all the stimuli.

For each stimulus in continuity experiments, we always compared two groups: with stimulus segments constituting one line or lying on two different lines (Fig. 6). The angle difference between lines varied from 90° to 23°. Unlike similarity experiments, we did not consider a 0° angle difference for continuity, because in that case two lines would be merged into one, and segments 1 and 4, 2 and 3 would coincide.

Since only two-group comparisons were conducted, we used a Wilcoxon rank test. For two smallest angle differences between lines and hence the greatest Gestalt-likeness (23°), we have not observed significant differences in synchrony. However, the greater angle difference and the lower Gestalt-likeness led to greater differences in synchrony. Significant differences were detected for greater angle differences: p < 0.05 for 45°, p < 0.001 for 68°, p < 0.01 for 90°. The 90° difference led to the second greatest significance level, and the results in general fell in line with our expectations: greater Gestalt-likeness corresponds to greater spike synchrony between segments.

There can be multiple reasons to explain the lower difference in synchrony between Gestalt-like and non-Gestalt combinations of segments (i.e. segments lying on the same or on different lines within one stimulus) for 90°. Since we measured the synchrony between segments 1 and 2, 3 and 4 (see Fig. 8 for continuity stimuli segmentation), one can observe that spatial distance is lower between 1 and 2 for 90°, than for all other angles. Thus, while the angle difference between segments increases, the spatial distance decreases at the same time. In accordance with the connectivity structure in V1, both spatial distance and orientation similarity are mapped on the interneuronal connectivity strength in our model (see Eqs. 1–5). Since the spike synchrony depends on connectivity (see Introduction), it depends on both spatial distance and orientation simultaneously. If the connectivity in V1 was organized differently and only incorporated the angle difference, then the spatial distance would not have an impact on the synchrony and thus the synchrony would be low for segments with 90° angle difference and higher for segments with smaller angle difference, regardless of the distance between them. Also, the continuity stimuli are more complex than similarity stimuli, despite the similar variety at the angle difference. Thus, the model receives more overall excitation, which may lead to increase in the system's response variability and unexpected results.

However, it is worth noting that the result of the Wilcoxon test still remains significant in this case. Also, we do not observe a significant difference in synchrony for the most Gestalt-like stimulus with the 23° angle difference, despite the greatest spatial distance between neurons. These two observations lead to the conclusion that synchrony still reflects the Gestalt structure of the continuity stimuli, although the effect is not as pronounced as for the similarity experiment.

We also emphasize that the synchrony differences in response to various Gestalt likeness were measured for the entire group of activated neurons, which means that the information about the stimulus is shared across all neurons receiving input. Thus, synchrony can operate as an emergent phenomenon encoding global information about the entire stimulus.

### Average pairwise synchrony and the Gestalt structure

**Figure 7:**
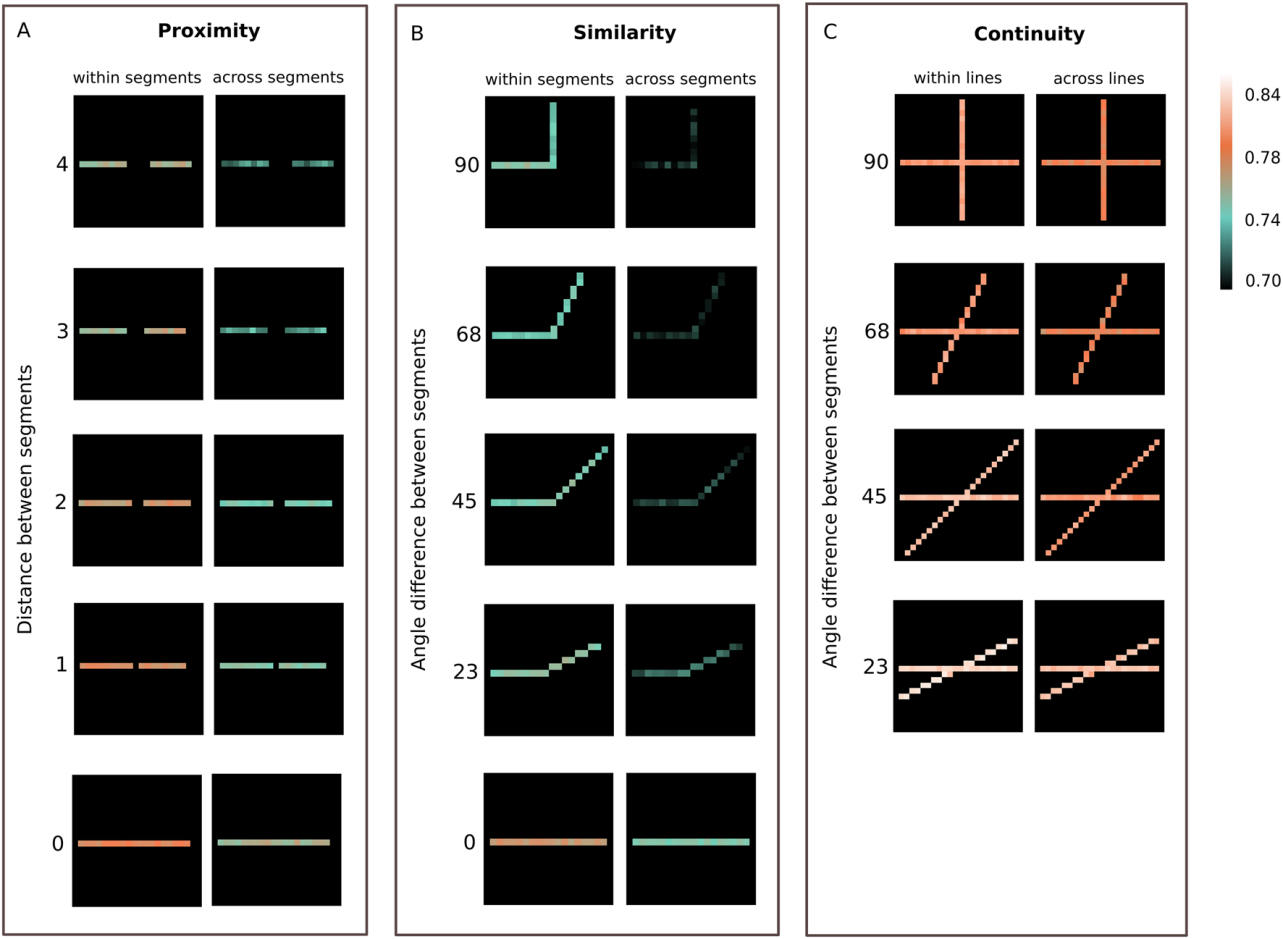
Avg pairwise Rsync. Avg pairwise Rsync measured for each stimulus separately. Only neurons receiving the external input are shown. Rsync of each neuron’s spike train with every neuron from the similar and the other segment is measured pairwise and then averaged. (**A**,**B**) Avg Pairwise Rsync measured for each stimulus separately, for proximity and similarity experiments. On the left, for each neuron the synchrony with the other neurons of its segment is measured. On the right, for each neuron of segment 1 the synchrony with the neurons of segment 2 is shown, and for each neuron of segment 2 the synchrony with the neurons of segment 1 is shown. (**A**) Avg Pairwise Rsync is demonstrated for stimuli with various distances between the segments. (**B**) Avg pairwise Rsync is demonstrated for stimuli with various angle differences between the segments. (**C**) Avg pairwise Rsync measured for each stimulus separately, for continuity experiments. On the left, for each neuron of segment 1 the synchrony with the neurons of segment 3 is shown, and for each neuron of segment 3 the synchrony with the neurons of segment 1 is shown. On the right, for each neuron of segment 1 the synchrony with the neurons of segment 2 is shown, and for each neuron of segment 3 the synchrony with the neurons of segment 4 is shown. Mean pairwise synchrony is demonstrated for stimuli with various angle differences between the lines.

**Figure 8:**
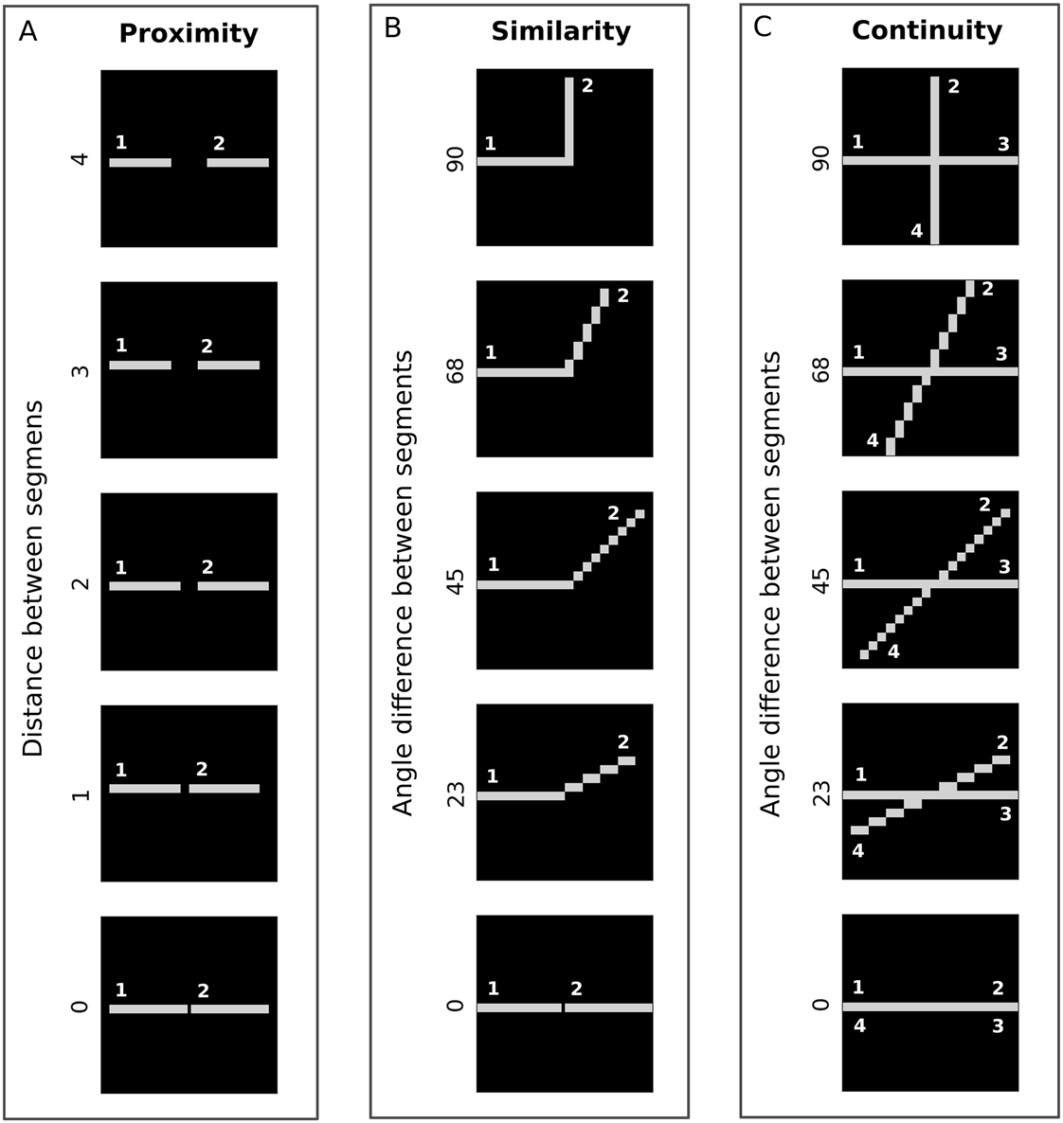
Input stimuli. Input stimuli for experiments in descending order of their adherence to Gestalt-likeness. (**A**) Input stimuli for the Gestalt principle of proximity. Each stimulus consists of two segments: 1 and 2. The spatial difference between segments varies from 4 to 0. (**B**) Input stimuli for the Gestalt principle of similarity. Each stimulus consists of two segments: 1 and 2. The angle orientation difference between segments varies from 90° to 0°. (**C**) Input stimuli for the Gestalt principle of continuity. Each stimulus consists of four segments: 1, 2, 3, 4. Segments 1 and 3, 2 and 4 constitute continuous lines. The angle difference between lines varies from 90° to 0°.

Figure 7 illustrates the difference between the Avg Pairwise Rsync within similar and different line segments. For all three types of experiments (proximity, similarity, and continuity), Rsync within one stimulus segment was higher than between neurons belonging to different segments. At the same time, Rsync both within one segment and between two segments was increasing in accordance with the Gestalt-likeness of the stimulus. Thus, the smaller the distance between two segments for proximity stimuli (see Fig. 8), the greater both within- and between-Rsync was. For the similarity and continuity stimuli, the smaller the angle difference between two segments, the higher the pairwise Rsync between them. Thus, the more similar the stimuli segments were to each other – the higher spike synchrony was observed between neurons tuned to different segments of these stimuli.

This illustrates that spike synchrony can be measured not only in a group, but also in a pairwise fashion, and still be an indicator of how closely the stimulus is following the Gestalt principles. Since the pairs for measurement included neurons within the stimulus on various distances from each other, this stays in line with the argument about the global nature of spike synchrony. It should also be noted that overall Avg Pairwise Rsync stays maximal for continuity experiments. Again, this observation is consistent with the Group Rsync increasing for continuity experiments due to the greater amount of both external and lateral excitatory input to neurons.

Additionally, we investigated how spike synchrony depends on the connectivity structure in the network, since synchrony in V1 was shown to arise from the horizontal intracortical connections. Avg Pairwise Rsync appeared to be correlated with the connectivity strength between neurons, with the Pearson r correlation coefficient up to 0.42. It shows that the connectivity structure is reflected in the patterns of synchronous firing to a certain extent. For more detail on correlation analysis please see Supplementary Note 2.

## Discussion

We showed that fully-excitatory spiking networks in a noisy environment demonstrate synchronous response to the Gestalt-like visual stimuli. The better stimuli follow the said Gestalt principles (proximity, continuity and similarity), the greater is spike synchrony. This demonstrates the computational role of spike synchrony as a mechanism for estimating the match between the stimulus and a prior encoded in a network connectivity structure.

Unlike a number of retinotopic modeling studies, our model does not incorporate inhibitory neurons; it is a fully-excitatory network. Although inhibitory neurons can induce zero-phase or near zero-phase synchronization pattern, we intentionally did not include them in the scope of this study, to investigate the pure effect of noise-induced coherence. This particular type of synchrony is shaped by the excitatory lateral connections and noise in the system, and we aimed to isolate noise-induced coherence from the other dynamics, caused by interactions between excitatory and inhibitory neurons. Reference^17^ illustrated that presence of the inhibitory neurons in the Izhikevich spiking system is not necessary for the emergence of noise-induced coherence, shaped by the network connectivity.

In V1, often co-activated excitatory cortical cells are well connected, which reflects the long-term visual experience. We argue that synchrony between such strongly connected units can serve as a measure of how well the perceived stimulus matches the acquired visual experience. Thus, its increase in response to Gestalt-like stimuli (unbroken lines, lines with the segments of similar orientation, etc.) shows that most often-experienced behaviorally relevant objects in the visual environment tend to follow the Gestalt structure. This is consistent with the natural statistics observations of^44,48–51^. That is, if the horizontal connections reflect the most often experienced visual objects and serve the basis for spike synchrony, it should be natural for neurons to synchronize in response to Gestalt-like stimuli.

From the Bayesian perspective, we demonstrated that synchrony in the model of V1 can be interpreted as a familiarity of an encoded stimulus, which is a match of prior probability of an input that is encoded in the network structure, and the stimulus that is encoded in the local spike rate of neurons. This mechanism is consistent with the observed spiking data recorded under stimuli conditions reflecting the Gestalt law^1^. As shown in Ref.^17^, synchrony can provide an estimate for match between a current stimulus and previously learned connectivity structure. Our model does not learn the connections directly, but instead uses a predefined connectivity matrix, which follows the connectivity rules typically observed in V1. We treat connections as previously learned, or pretrained, and calculate the match of connectivity with input stimuli through measuring spike synchrony in the system. This mechanism could equally be utilized in models which would actively learn network connections different from V1 connectivity.

Alternatively, spike synchrony can be viewed in the light of contour integration, which was previously studied in Ref.^12^ with a similar Izhikevich spiking model. Contours in natural image statistics can often be seen as smoothly connected elements, which is in line with the idea of synchrony reflecting commonly occurring natural image statistics through V1 connectivity structure. However, when interpreting spike synchrony in an Izhikevich network, one should keep in mind that in multi-layer models the absolute values of synchrony can also be influenced by the Izhikevich model parameters^55^.

The transition of a strictly local encoding of stimulus features, towards an emergent property that forms spatially extended representation based on spike synchrony, is a crucial and efficient computational feature. It allows separate processing of the stimulus content on the local level, and processing based on global properties linked to stimulus familiarity. However, we emphasize that both local and global properties are essential to fully characterize the stimulus. Two stimuli can be equally familiar to the system, and yet very different from one another. For example, consider one stimulus with a high Gestalt proximity and low Gestalt similarity, and another – with high similarity and low proximity. Both stimuli would evoke similar synchrony in the model, and only consideration of the local properties, such as active neurons receptive fields, could provide information necessary to identify the stimulus. Global synchrony does not replace local features, but rather provides additional information.

It can be, for instance, a proxy for how well a stimulus is encoded (low familiarity corresponds to weak information and vice versa), or whether the stimulus is new or has been encountered before, regardless of what exactly the stimulus is. For agents, animals and humans, this information might be at least as relevant as the stimulus information itself. This emergent proxy will allow the agent to plan and perform continuous actions that can be stopped if the collected information is sufficient. Such active sampling and inference is discussed in different aspects across the fields of cognitive science, neuroscience and machine learning, and seems to be best described by the theory of Posterior sampling in reinforcement learning^56^. We therefore believe that spike synchrony, as an emergent phenomenon of spiking neural networks with strictly local stimulus feature encoding, is a computational principle that allows for the inference of global features, i.e. stimulus family, that can be relevant for efficient active learning and sensing.

## Methods

### Input stimuli

In our study, we examined the relationship of spike synchrony and the binding of visual stimulus components for three Gestalt grouping cues: proximity, similarity, and continuity^45^. The principles can also interact with each other, either strengthening or weakening their effects when combined.

1. Proximity principle: According to Gestalt theory, the principle of proximity states that when single visual components are located near each other, they are perceived as forming a larger visual composition. The aggregation of individual elements is grouped together to create a new superordinate entity.
2. Similarity principle: Another principle of Gestalt theory is the similarity principle, which involves integrating visual components into perceptual groups based on the similarity of their appearance. This principle classifies elements according to their visual features.
3. Continuity principle: The continuity principle, a fundamental aspect of Gestalt theory, organizes patterns into new compositions based on the alignment of their established direction. Conversely, patterns with abrupt changes in orientation are partitioned into distinct elements. For intersecting lines, the concept of Gestalt-likeness relates to how effectively the lines form a continuous shape or pattern. The continuity principle helps distinguish stimuli with visual overlap.

For the experiments, we ran simulations with a spiking neural network receiving artificial stimuli. The visual input for our network was generated from simple artificial images of size 20 by 20 pixels. These stimuli represent the input for the early stages of the visual pathway, constituting simple cells recognizing edges with location and orientation selectivity and implementing the examples of Gestalt theory principles (1)—(3) respectively. Before being presented to the network, input stimuli are partitioned into different channels by an edge detector sensitive to orientation. For the edge detector, 2-dimensional kernels are convolved over the input stimuli to generate 5 distinct channels fed into the network (see Supplementary Figs. 1–3). This reflects the selectivity of the visual cortex to edges of certain angles through hypercolumns and orientation columns. Edges in the input stimuli are undirected, limiting the relevant range of orientations from 0° to 180°. Since the size of our networks grows exponentially with the number of orientation channels, for reasons of computational efficiency, in the experiments only input stimuli with an orientation between 0° to 90° were considered. Sensitivity to the degree of orientation was set to 22.5°, resulting in 5 channels for input stimuli of orientations [0°, 23°, 45°, 68°, 90°].

Additionally, we introduced background activity in the form of uniform noise that is added to the input stimulus at every simulation step, simulating real-world conditions where input signals are subject to fluctuations. The signal-to-noise ratio was set to 0.4, with any higher ratio resulting in the onset of runaway excitation. During runaway excitation, the activity within the network is only limited by the spike-rate saturation leading to the constant firing of neurons without any meaningful modulation or information processing.

The stimulus is transformed into spiking activity based on a Poisson model of spike generation at a rate of 40 Hz with a temporal resolution of 0.5 ms. The network receives this probabilistic input from the Poisson spiking process—a mathematical model for generating random events that occur independently of each other over time with a constant average rate of occurrence. Creating spike patterns probabilistically allows us to capture the stochastic nature of neural activity.

A new set of stimuli patterns was created for each experiment, each reflecting a range of Gestalt-likeness of the experiment’s Gestalt principle respectively (Fig. 8). The effective representation of the range of degrees in Gestalt-likeness closely follows the definition of Gestalt principles, with the assumption that our stimuli models similar patterns present in the early processing of the visual pathway sufficiently close.

Input stimuli for the Gestalt principle of proximity are based on the spatial distance between segments. The closer the two segments are, the more likely they will be perceived as a group. As the distance between segments increases, so does the likelihood of perceiving them as separate entities. Therefore, the degree of Gestalt-likeness of stimuli decreases with an increase of range between two segments. A set of 5 stimuli with between-segments spatial distances varying from from 0 to 5 pixels, was used in proximity experiments (Fig. 8). Note that the size of stimulus segments varies from stimulus to stimulus. Our design choice was to let it vary, but fix the entire stimulus size and keep stimuli smaller, for the performance efficiency. The size of stimulus segments did not have an impact on the results (see Supplementary Fig. 5).

Input stimuli for the Gestalt principle of similarity are based on the orientation similarity, the similarity of segments’ degree of rotation. The closer two segments are in visual appearance the higher their Gestalt-likeness. Segments with minimal orientation differences are perceived as having high orientation similarity and are more likely to be perceived as a group. As the difference in orientation between segments increases, making them visually more distinct, so does the likelihood of perceiving them as separate segments. Therefore, the degree of Gestalt-likeness of stimuli decreases. A set of 5 stimuli with 90°, 68°, 45°, 23° and 0° between-segments orientation differences increasing in their Gestalt-likeness, was used in similarity experiments (Fig. 8).

Input stimuli for the Gestalt principle of continuity are based on the abruptness of a segment’s change of direction in two intersecting lines. Elements arranged in a continuous manner are more likely to be perceived as belonging together. Segments with minimal orientation differences are perceived as having high orientation similarity and are more likely to be perceived as a continuous line. Additionally, as the difference in orientation between two lines decreases so does the likelihood of perceiving the line’s segments as a disjoint part that belongs to the other’s segment instead. Therefore, a smaller difference indicates that the stimulus follows the continuity principle more strongly. A set of 4 stimuli with 90°, 68°, 45°, and 23° between-lines orientation differences increasing in their Gestalt-likeness, was used in continuity experiments (Fig. 8). Unlike in the similarity experiments, a 0° angle difference for continuity was not considered for the experiments, since here both lines fully merge into one, and segments 1 and 4, 2 and 3 coincide.

All parameters used for generating input stimuli can be found in Supplementary Table 5.

### Connectivity

A network architecture was designed to reflect the retinotopic organization of the visual cortex with artificial spiking neurons. It comprises a single n × m layer of Izhikevich neurons where n, m correspond to the stimulus dimensions. The fixed connectivity of the network structure models the retinotopic organization of the visual cortex (V1) and is organized as hypercolumns of neurons selective towards the location and the orientation of stimuli. Specifically, the connection strength between neurons is determined by two factors: the spatial proximity of the neurons' receptive fields and the similarity in the angle orientation of the stimuli (see Fig. 1).

1. Spatial proximity: Each neuron is receptive to location-specific input from both external stimuli matching their retinal coordinates and horizontal connections by all other neurons with their connection strength inversely proportional to their distance (Eq. 2). In this way, neighboring neurons with similar receptive fields will receive stronger input than distant ones of dissimilar receptive fields. As a distance metric, we used the Chebyshev distance (L_∞_), which quantifies the maximum difference between corresponding components of two vectors in a multi-dimensional space. The distance between two points, A(x_1_, y_1_) and B(x_2_, y_2_), in a two-dimensional space is here defined as the maximum absolute difference between their corresponding coordinates along the x and y axes (Eq. 1). Compared to Euclidean distance, the Chebyshev distance is invariant to the orientation of vectors, treating vectors with horizontal, vertical, and diagonal directions equally.
  1 $$L_{\infty } (x,y) = max(\left| {x_{2} - x_{1} } \right|,\left| {y_{2} - y_{1} } \right|)$$
  2 $$W^{d} = 1/(L_{\infty } + 1)$$
2. Orientation similarity: Neurons within the network are also selective to the orientation of stimuli, which is manifested in the formation of orientation-selective cortical columns. A subnetwork of neurons that receive input from the same region but select for different orientations represents an orientation-selective hypercolumn. All neurons in the network are connected to each other, with a connection strength that is inversely proportional to the angle difference (Eq. 4).
  3 $$\Delta_{\alpha ,\beta } = 90^{ \circ } - ||\alpha - \beta | - 90^{ \circ } |$$
  4 $$W^{a} = 1/(\Delta_{\alpha ,\beta } + 1)$$

Here, *α* and β stand for the angles which two neurons forming the connection are sensitive to.

Connection weights W^d^ and W^a^ are scaled with scaling factors for the spatial differences S^d^ and angle differences S^a^ respectively. Later, the resulting connection weights are scaled with a factor S^lat^ and form the resulting lateral connectivity matrix W^lat^.

5 $$W^{lat} = (W^{d} \times S^{d} + W^{a} \times *S^{a} ) \times S^{lat}$$

By taking both spatial distance and orientation similarity into account for building a connectivity matrix, our proposed network model emulates a simple retinotopic organization of V1 and provides a mechanism for the representation of location and orientation-specific stimuli through the connectivity of neurons within the network. Importantly, spatial proximity and orientation similarity have an equal impact on the overall connection strength. No self-connections or inhibitory connections were used in our network.

All parameters used for building a connectivity matrix can be found in Supplementary Table 8.

### Model

To simulate neurons, we used the Izhikevich model for spiking neural networks^54^ with excitatory pulsed coupling. The Izhikevich model of a spiking neuron emulates the dynamics of the classical Hodgkin-Huxley model^57^ but operates more efficiently. It is a two-dimensional system, where each neuron is characterized by two internal variables: the membrane potential v_i_ and the recovery variable u_i_. In our network only excitatory connections are used (see “Discussion”).

6 $$\dot{v}_{i} = 0.04v_{i}^{2} + 5v_{i} + 140 - u_{i} - I_{i} + \epsilon$$

7 $$\varepsilon = (r - 0.5) \times S_{\varepsilon }$$

8 $$\dot{u}_{i} = a(bv_{i} - u_{i} )$$

9 $${v_{i} \leftarrow c}$$

10 $$u_{i} \leftarrow u_{i} + d$$

Equations (6) and (8) describe the dynamics of a single neuron i. Its voltage v_i_ and recovery u_i_ change over time. In Eq. (6), the parameter I_i_ defines the entire input to the neuron i. It is further described in Eq. (11), (12), (13) ε stands for the voltage noise, and Eq. (7) shows how it is formed: r is a random number taken from a uniform distribution between 0 and 1, and modified with a voltage noise scaling factor S_ε_ set to 0.3.

In Eq. (8), a stands for the timescale of u_i_: the bigger it is, the faster is recovery. In our model, we consider a = 0.02. The parameter b describes the sensitivity of the recovery variable u_i_ to fluctuations of the membrane potential v_i_. We set b = 0.2.

Equations (9), (10) show how the afterspike dynamics of a neuron i. When the value of v_i_ exceeds the activation threshold 30 mV, we record a spike event. After the spike is detected, the value of v_i_ is reset according to Eq. (9), and the value of u_i_ is updated according to Eq. (10). The voltage reset constant c is set to − 65 mV, and the recovery update variable d is set to 6.

The parameter I_i_ defines the entire input to the neuron i. It consists of input currents received from lateral connections (I^lat^) and the external stimulus (I^ext^). The evolution of these currents follows the nonlinear chemical model: it describes the faction of open receptors in a synaptic connection, which is driven by a neurotransmitter concentration, which in turn depends on the incoming spikes, either lateral or external.

11 $$I_{i} = I_{i}^{lat} + I_{i}^{ext}$$

12 $$I_{i}^{lat} = (v_{i} - E) \cdot \sum\limits_{j \in J(i)} {W_{ij}^{lat} } \times r_{j}$$

13 $$I_{i}^{ext} = (v_{i} - E) \cdot r_{i}^{ext} \times S^{ext}$$

Here, J(i) is a set of neurons connected to a neuron i. W^lat^ is a lateral connectivity matrix (see "Methods" "Connectivity"), where each W_ij_ represents conductance of a synaptic connection between neurons i and j. Thus, the lateral input current is represented by a sum of individual input currents from all neurons forming lateral connections with the neuron i, weighted by a conductance of each connection.

There is no external connectivity matrix, because each neuron receives external input simply as an independent Poisson spiking process, scaled by a factor S^ext^. The firing rate of a Poisson process is set to 40 Hz (see “Methods” "Input stimuli"). E stands for synaptic reversal potential.

Now, the conductance of every synaptic connection is adjusted by the open receptor fraction r, influenced by incoming spikes. For both external and lateral connections, r is driven by neurotransmitter concentration in the synaptic cleft [T]_j_, which in turn is represented by a pulse of duration τ = 0.02 after each incoming spike.

14 $$\dot{r}_{j} = \alpha \left[ T \right]_{j} (1 - r_{j} ) - \beta r_{j}$$

15 $$\left[ T \right]_{j} = \Theta (T_{j} + \tau - t)\Theta (t - T_{j} )$$

r_i_ is parameterized, also for both external and lateral connections, by the rise and decay time constants α = β = 8. The transmitter concentration [T]_j_ is the product of two heaviside step functions Θ. They define that neurotransmitter is present in the synaptic cleft ([T]_j_ = 1) starting from the occurrence of a presynaptic spike at the moment T_j_, until the moment T_j_ + τ.

All model parameters can be found in Supplementary Table 6, and simulation parameters in Supplementary Table 7.

### Simulations

Separate experiments for the three Gestalt principles were conducted, each with an individual set of stimuli generated as described above. Our simulations were run for a total duration of 1000 ms at a temporal precision in the model of 0.005 ms, with an additional initial transient period of 500 ms. This transient period was added at the initial phase of the simulation during which the network's state has not yet fully stabilized into its steady-state behavior. This interval ensures that any synchronous activity as a result of the network’s starting conditions will have dissipated, and subsequent synchronous activity is only caused by the input stimulus and the network’s connectivity. The activity during the transient period is excluded from the data analysis. For the analysis of the spike trains the data were downsampled to 0.5 ms.

### Measure synchrony

We measure the temporal synchrony of the neuron spike traces following the procedure from Korndörfer et al. 2017 by adopting the Rsync metric (Eq. 16)^17^. It computes the average degree of zero-lag synchrony of the network, which ranges from 0 with all neurons firing out of phase, to 1 with all neurons strictly simultaneously firing.

16 $$R_{{sync}} \left( {S,\,T} \right) = \frac{{\widehat{{Var}}\left[ {\left\langle {A_{i} \left. {\left( t \right)} \right\rangle _{{i \in S}} } \right.} \right]_{{t \in \,T}} }}{{\left\langle {\widehat{{Var}}\left. {\left[ {A_{i} \left( t \right)} \right]_{{t \in \,T}} } \right\rangle _{{i \in S}} } \right.}}$$

Here, ${A}_{i}$ is an activation trace of the neuron $i$ from the population $S$. To retrieve this activation trace, the raw binary spike train is first convolved with a causal exponential kernel k(t) = e^–2t^, with the timescale = 3 ms. In related studies aimed at measuring joint activity of multiple spiking neurons, the timescale varies between 1 and 10 ms^58^. Our chosen timescale falls in this range and is comparable to the one of an EPSP^58^. The precise choice of a timescale does not impact the results, see Supplementary Fig. 4 for details.

Synchrony of a neuron population S is measured on a time interval $T$. It is given by the variance of the mean activation trace of this population, divided by the average variance of all neurons in the population. Intuitively, when all neurons are perfectly synchronous, i.e. fire precisely at the same time, the variance of their average activation trace is equal to a variance of each individual neuron and, respectively, to their average variance.

In the synchronized case, the sum of signals will display large-amplitude oscillations, while in the unsynchronized case, the individual signals will be out of step with each other and their sum will be nearly constant. Due to the sensitivity of the measure to the number of active neurons, subpopulations of the network are drawn from neurons that receive external input from the stimulus segment. The neurons drawn can be either from the same stimulus segment for average within-stimulus synchrony or across stimulus segments for average between-stimulus synchrony.

### Equipment and settings

Figures 1, 2 were produced using Google Slides web application. Figures 3, 4, 5, 6, 7 and 8 were produced programmatically, with use of Python scripts available on the Github repository of the project. Plots for each experiment were produced separately, later they were combined into a single Figure using an Inkscape-0.92.3- × 64 application for Windows. The titles were also added to the resulting figures with use of Inkscape.

### Supplementary Information

Supplementary Information.

## Data Availability

All data in the work was programmatically generated. The code for generating data, building a model, running simulations and analyzing the simulation results, as well as the simulated data logs and statistics, are publicly available on a GitHub repository at https://github.com/rainsummer613/synchrony. Additionally, we assigned a DOI to the repository via Zenodo: 10.5281/zenodo.10145353.
