# Supplementary material for: Spike synchrony as a measure of Gestalt structure
Source: Sci Rep. 2024 Mar 11;14:5910. doi: 10.1038/s41598-024-54755-w (PMC10928224; doi:10.1038/s41598-024-54755-w)
Supplement: Supplementary file 1 — Supplementary Information. [file 41598_2024_54755_MOESM1_ESM.pdf]

Spike synchrony as a measure of Gestalt structure

Viktoria Zemliak[1¶*], Julius Mayer[1¶], Pascal Nieters[1], Gordon Pipa[1]

[1]Institute of Cognitive Science, University of Osnabrück, Osnabrück, Germany

*Corresponding author

E-mail: vzemliak@uos.de (VZ)

[¶]These authors contributed equally to this work.

Supplementary

Supplementary Figure S1

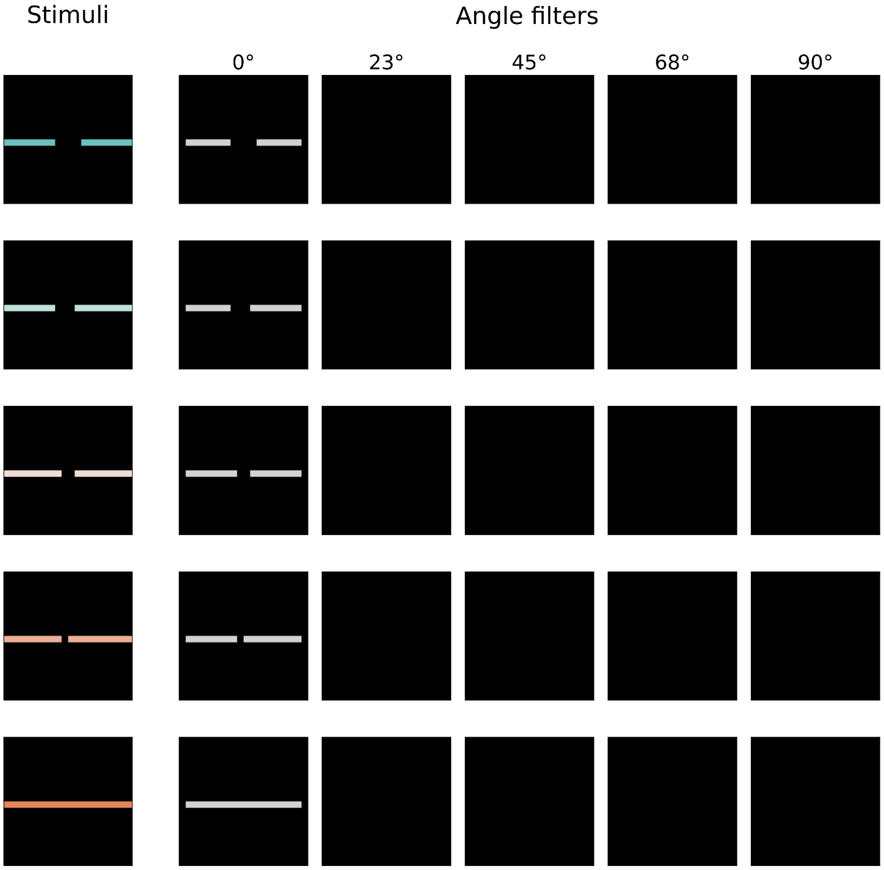

Figure S1. Angle detection for proximity stimuli

All stimuli for the proximity experiments, with corresponding angle maps showing the results of angle detection. Each map illustrates the activity of a group of neurons with receptive fields sensitive to a specific angle.

Supplementary Figure S2

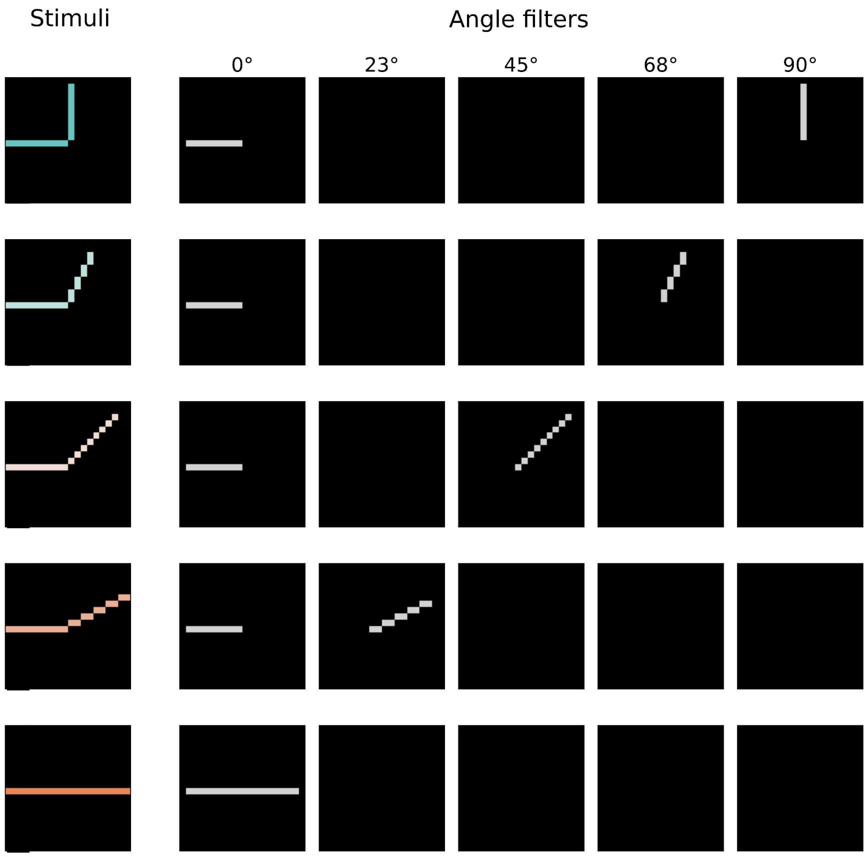

Figure S2. Angle detection for similarity stimuli

All stimuli for the similarity experiments, with corresponding angle maps showing the results of angle detection. Each map illustrates the activity of a group of neurons with receptive fields sensitive to a specific angle.

Supplementary Figure S3

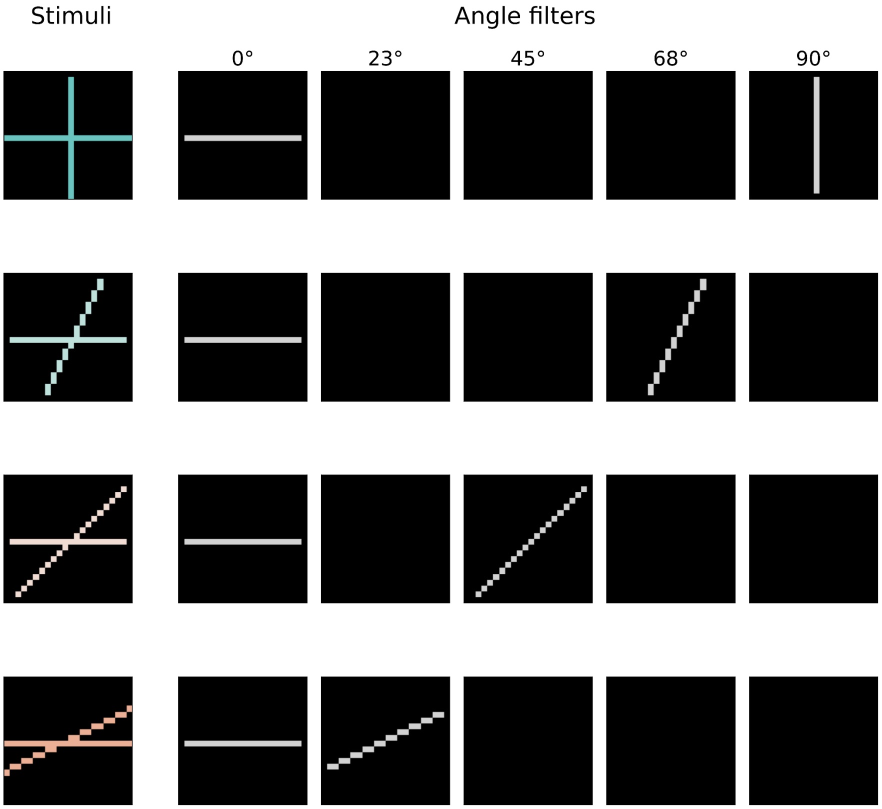

Figure S3. Angle detection for continuity stimuli

All stimuli for the continuity experiments, with corresponding angle maps showing the results of angle detection. Each map illustrates the activity of a group of neurons with receptive fields sensitive to a specific angle.

Supplementary Figure S4

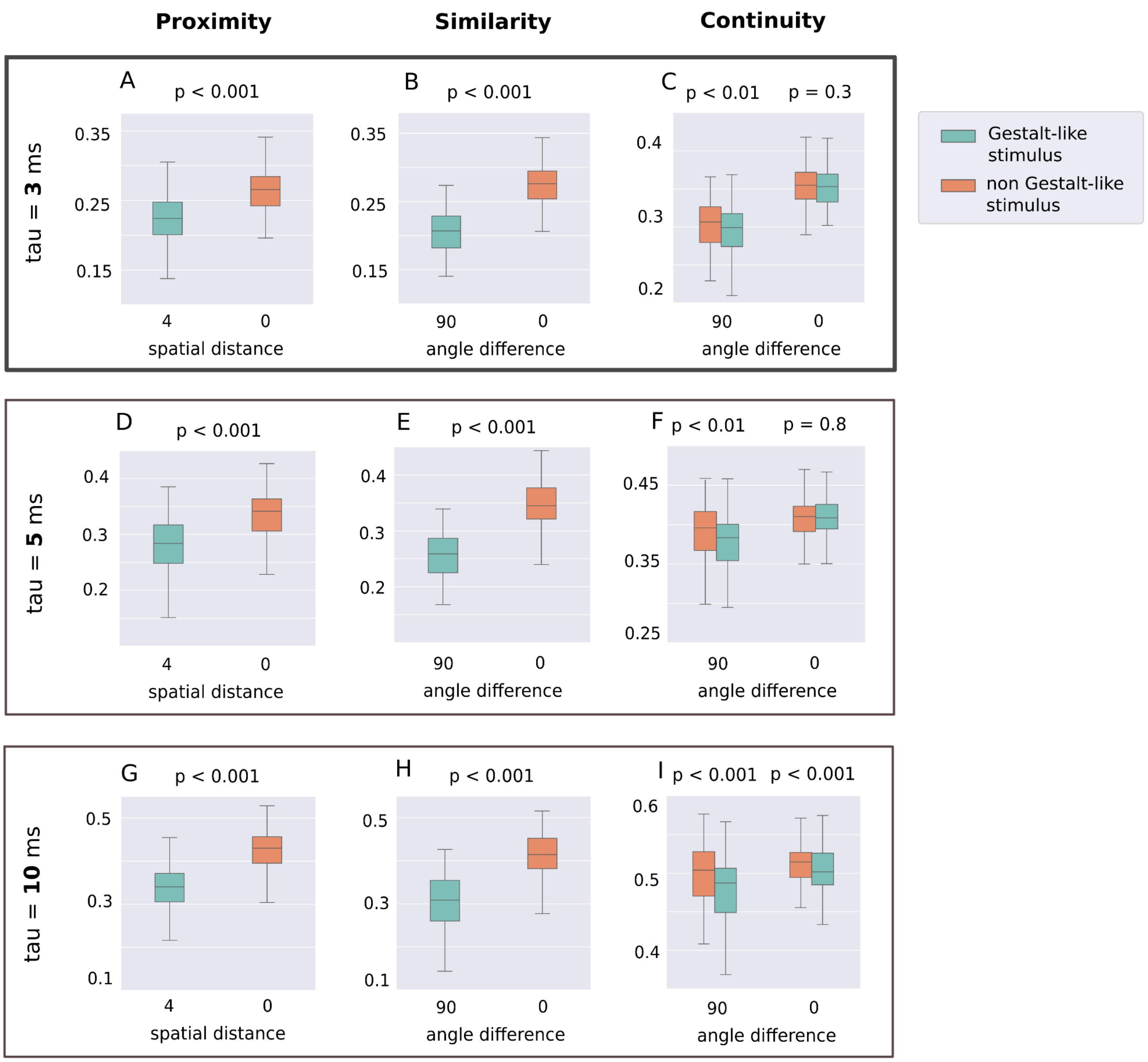

Figure S4. Group Rsync measured with various exponential timescale

Comparison of group Rsync for all experiments, with a different exponential timescale, applied to spike traces before computing Rsync. **A-C**. Measurements with the timescale = 3 ms, used to produce all experimental results in the main text. **D-F**. Measurements with the timescale = 5 ms, the significance levels are similar to A-C. **F-I.** Measurements with the timescale = 10 ms, the significance levels mostly similar to A-C.

Supplementary Figure S5

A

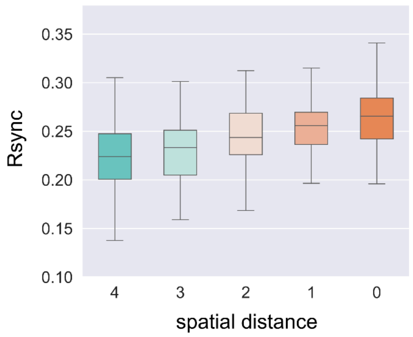

B

spatial distance	4	3	2	1	0
**4**	1	1	**0.0006***	**0***	**0***
**3**	1	1	0.05	**0.0001***	**0***
**2**	**0.0006***	0.05	1	0.85	0.001**
**1**	**0***	**0.0001***	0.85	1	0.3
**0**	**0***	**0***	0.001**	0.3	1

C

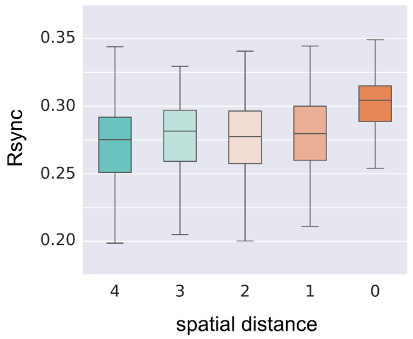

D

spatial distance	4	3	2	1	0
**4**	1	0.92	1	**0***	**0***
**3**	0.92	1	1	0.33	**0***
**2**	1	1	1	1	**0***
**1**	**0***	0.33	1	1	**0***
**0**	**0***	**0***	**0***	**0***	1

Figure S5. The comparison of Rsync for two types of proximity stimuli

Rsync values for two types of the proximity experimentю **A-B**. Rsync for stimuli with a varied segment size, but fixed stimuli size. **C-D**. Rsync for stimuli with a fixed segment size and varied stimulus size.

Supplementary Note 1. Synchrony of time-jittered spikes

Throughout the experiments we hypothesized that spike synchrony provides an additional information crucial for recognizing stimulus familiarity, and it highly relies on the temporal structure of the spike trains. To test whether the spike synchrony really depends on this fine temporal structure and not on e.g. firing rate, we did a control

measurement for all three experiments with group synchrony. For each spike train, spike times were randomly jittered across the time scale: each spike time was randomly shifted in time within the range of (-80; +80) ms from its actual spiking. Then Rsync was measured on such modified spike trains with destroyed temporal structure (Fig. S6).

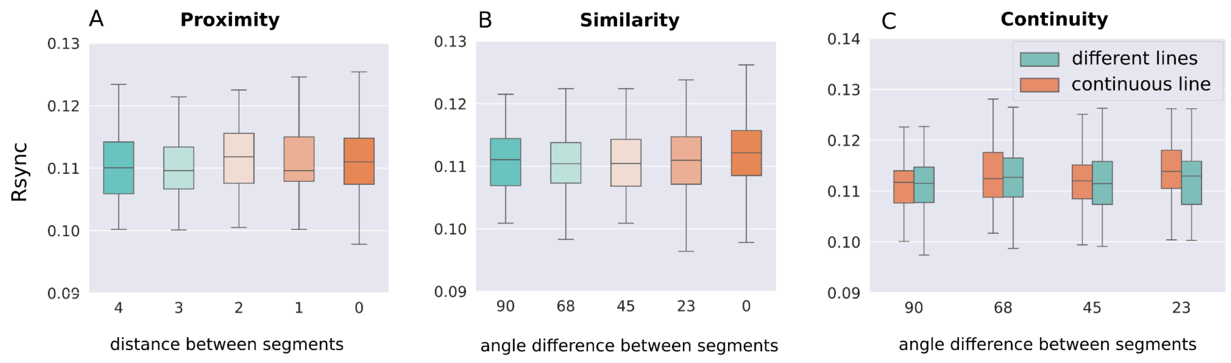

Figure S6. Group Rsync between segments for time-jittered spikes

Comparison of group Rsync for all stimuli conditions on time-jittered spikes. For every stimulus, 5 neurons were randomly selected from each stimulus segment, and the group Rsync was measured between all 10 of them. **A.** Comparison of group Rsync for proximity stimuli with various distances between the segments. **B.** Comparison of group Rsync for similarity stimuli with various angle differences between the segments. **C.** Comparison of group Rsync for continuity stimuli with various angle differences between the lines constituting a stimulus.

In contrast with the original experiments with non-jittered spike trains (see Results), for jittered spike trains synchrony does not change depending on the Gestalt structure of the stimulus. Not only no significant differences between experimental conditions are observed, but also the overall synchrony level is much lower than for the original non-jittered spike data. This holds true for all three Gestalt experiments. Thus, the temporal structure of spike trains contains synchrony information which is crucial for

detecting Gestalt structure and hence, in our setting, prior probability of the input stimulus.

We applied similar statistical tests to time-jittered spike trains, as to the original spike data. For the proximity experiments, Kruskal-Wallis test showed a non-significant result with the effect size = 0.005 and p-value = 0.17, while the same test on the non-jittered data revealed significant between-group differences with the effect size = 0.16 and p-value < 0.001. We further applied a nonparametric Dunn test with Bonferroni adjustment for multiple comparisons (Table S1). It found no significant differences in synchrony between any stimuli.

Table S1. Dunn test for proximity experiments with time-jittered spike trains

spatial distance	0	1	2	3	4
**0**	1	1	1	1	1
**1**	1	1	1	1	1
**2**	1	1	1	0.43	0.41
**3**	1	1	0.43	1	1
**4**	1	1	0.41	1	1

The results of Dunn test with Bonferroni correction for groups with various spatial distances between stimulus segments for time-jittered spike trains, rounded to 2 decimal.

For time-jittered data from the similarity experiments, Kruskal-Wallis test did not find any significant between-group differences with the effect size = 0.007 and p-value = 0.12, whereas the effect size = 0.38 and p-value < 0.001 for non-jittered data. The subsequent non-parametric Dunn test on time-jittered data confirmed the absence of any differences in synchrony between stimuli with various angle differences (Table S2),

although for the original non-jittered data Kruskall-Wallis test with Dunn post hoc test revealed multiple significant differences.

Table S2. Dunn test for similarity experiments with time-jittered spike trains

**angle difference**	**0**	**23**	**45**	**68**	**90**
**0**	1	0.66	0.17	0.33	0.36
**23**	0.66	1	1	1	1
**45**	0.17	1	1	1	1
**68**	0.33	1	1	1	1
**90**	0.36	1	1	1	1

The results of Dunn test with Bonferroni correction for groups with various angle differences between stimulus segments for time-jittered spike trains.

For continuity experiments with time jittering, we again followed the procedure for the original spiking data and compared two groups: with stimulus segments constituting one or two different lines. The angle difference between lines varied from 90° to 23°. We applied a Wilcoxon rank test and have only revealed a slightly significant result for the difference 23° (Table S3), unlike when measuring synchrony on the original data.

Table S3. Wilcoxon rank test for continuity experiments with time-jittered spike trains

**angle difference**	**Wilcoxon p**
**90**	0.63
**68**	0.35
**45**	0.73
**23**	**0.02***

The results of the Wilcoxon rank test for groups with various angle differences between stimulus segments for time-jittered spike trains, rounded to 2 decimal. * stands for $p < 0.05$.

Therefore, the statistical tests on time-jittered data from all three experiments showed no to very few statistically significant between-group differences. We observed no consistent pattern of significant differences depending on Gestalt structure of the stimuli across the experiments. Thus, the Gestalt structure and hence the prior probability of the stimulus can indeed be derived from the temporal structure of the data, which is captured by synchrony. When this fine temporal structure is destroyed, it is no longer possible to retrieve this information via our synchrony measure.

Supplementary Note 2. Avg Pairwise Rsync reflects the connectivity structure

In V1, spike synchrony arises from the horizontal intracortical connections [26-28]. To show how it depends on the connectivity structure in the model, we visualized matrices of connections between neurons and pairwise synchrony matrices next to each other (Fig S7). Only neurons receiving the external input were considered.

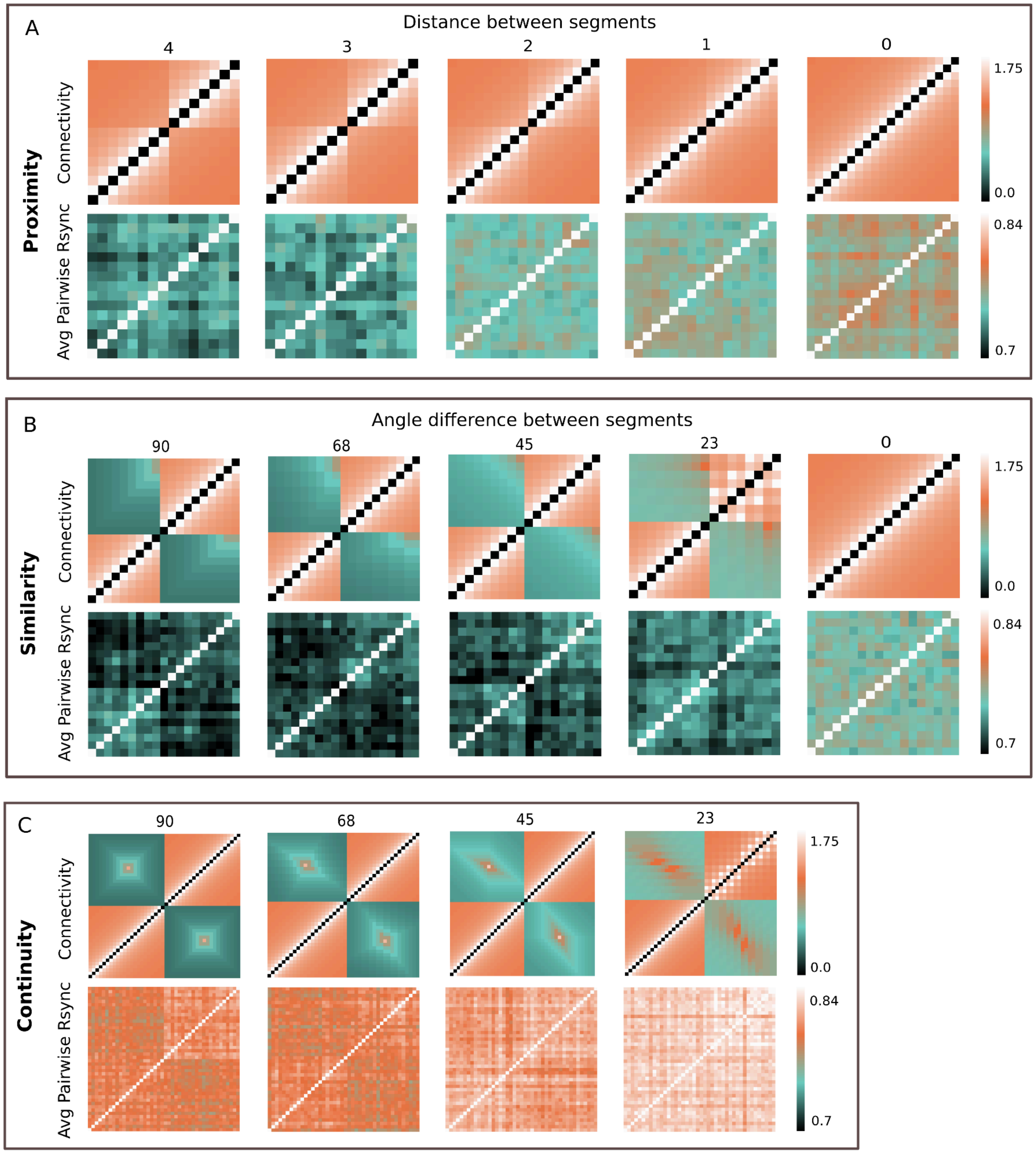

Figure S7. Rsync & connectivity

Comparison of the connectivity matrix and Avg Pairwise Rsync values for a group of neurons receiving the external input. **A.** Connectivity matrices and corresponding Avg Pairwise Rsync maps for proximity experiments. **B.** Connectivity matrices and corresponding Avg Pairwise Rsync maps for similarity experiments. **C.** Connectivity matrices and corresponding Avg Pairwise Rsync maps for continuity experiments.

What we found is that the connectivity structure was to a certain degree reflected in Avg Pairwise Rsync values. First, in all experiments the higher overall connectivity strength was leading to higher synchrony. Second, the structure of the connectivity matrix was reflected in the structure of the pairwise synchrony matrix to a certain degree. The structure of the synchrony matrix was able to capture the most pronounced structural details of the connectivity matrix. For example, the alterations of high and low connectivity values for proximity weight matrices were also visible in the corresponding pairwise synchrony matrices.

To quantify the similarity between connectivity and Avg Pairwise Rsync, we measured the cross-correlation between two matrices for all experimental conditions, averaged over 100 trials each (Table S4).

Table S4. Correlation between connectivity & pairwise Rsync

Proximity		Similarity		Continuity	
**distance**	**Pearson r ± sd**	**angle difference**	**Pearson r ± sd**	**angle difference**	**Pearson r± sd**
4	0.41 ± 0.04	90°	0.33 ± 0.06	90°	0.24 ± 0.07
3	0.39 ± 0.05	68°	0.32 ± 0.02	68°	0.23 ± 0.06
2	0.39 ± 0.04	45°	0.32 ± 0.07	45°	0.24 ± 0.12
1	0.39 ± 0.04	23°	0.33 ± 0.04	23°	0.2 ± 0.1
0	0.37 ± 0.04	0°	0.38 ± 0.04	0°	

Pearson r correlation coefficients between Connectivity and Avg Pairwise Rsync, rounded to 2 decimal. P-values are < 0.005 for all experiments.

What we found is that measured Pearson r values exceeded 0.3 in most cases, which implies a positive correlation. The correlation was the strongest for proximity experiments, likely due to neurons always sharing the same angle orientation and thus having a more homogeneous connection weights distribution. In contrast, the connectivity in continuity experiments tends to form more complex patterns, due to the more complex stimuli. It seemed to influence the correlation strength, which was lower than the average correlation strength for proximity and similarity experiments. For all the experiments spike synchrony was moderately or weakly correlated with the interneuronal connectivity structure.

Supplementary Table S5

**Parameter**	**Value**	**Units (where applicable)**
Stimulus width	20	pixels
Stimulus height	20	pixels
Angle resolution	22.5	degrees
Input firing rate	40.0	Hz
Input noise amount	0.4	
Input noise type	uniform	
Input strength	2.2	

Supplementary Table S6

**Parameter**	**Value**	**Units (where applicable)**
Simulation time step	0.005	ms
Initial membrane potential	30	mV
Initial recovery variable	30	mV
Izhikevich a (time scale of recovery variable)	0.01	
Izhikevich b (sensitivity of recovery variable)	-0.1	
Voltage afterspike reset	-65.0	mV
Recovery afterspike update	12.0	mV
Excitatory reverse potential	0.0	mV
After-spike neurotransmitter presence in lateral connections	0.02	ms
After-spike neurotransmitter presence in external connections (from input to model neurons)	0.02	ms
Rising time of open receptors in lateral connections	8.0	ms
Rising time of open receptors in external connections	8.0	ms
Decay time of open receptors lateral connections	8.0	ms
Decay time of open receptors in external connections	8.0	ms
Maximum transmitter concentration in lateral connections	1.0	
Maximum transmitter concentration in external connections	1.0	
Voltage noise amount	0.3	

Supplementary Table S7

**Parameter**	**Value**	**Units (where applicable)**
Simulation length	2000	ms
Downsampling	100	timesteps

Supplementary Table S8

**Parameter**	**Value**	**Units (where applicable)**
Spatial distance weight	0.5	
Orientation difference weight	0.5	
Lateral connection weight	2.2	
